# Disseminated Emergomycosis in a Person with HIV Infection, Uganda

**DOI:** 10.3201/eid2509.181234

**Published:** 2019-09

**Authors:** Isabelle Rooms, Peter Mugisha, Thilo Gambichler, Eva Hadaschik, Stefan Esser, Peter-Michael Rath, Gerhard Haase, Dunja Wilmes, Ilka McCormick-Smith, Volker Rickerts

**Affiliations:** University Duisburg-Essen, Essen, Germany (I. Rooms, E. Hadaschik, S. Esser); Mbarara Referral Hospital, Mbarara, Uganda (P. Mugisha);; St. Joseph Hospital Bochum, Bochum, Germany (T. Gambichler);; University Hospital Essen, Essen (P.-M. Rath);; RWTH Aachen University Hospital, Aachen, Germany (G. Haase);; Robert Koch Institute, Berlin, Germany (D. Wilmes, I. McCormick-Smith, V. Rickerts)

**Keywords:** Emergomycosis, Broad-range fungal PCR, Formalin-fixed paraffin-embedded biopsy, FFPE, Fungi, HIV/AIDS and other retroviruses, Uganda

## Abstract

We describe emergomycosis in a patient in Uganda with HIV infection. We tested a formalin-fixed, paraffin-embedded skin biopsy to identify* Emergomyces pasteurianus* or a closely related pathogen by sequencing broad-range fungal PCR amplicons. Results suggest that emergomycosis is more widespread and genetically diverse than previously documented. PCR on tissue blocks may help clarify emergomycosis epidemiology.

Emergomycosis is a fungal infection caused by fungi of the newly described genus *Emergomyces*, of the order Onygenales, which includes obligate fungal pathogens, such as *Histoplasma*, *Blastomyces*, and *Paracoccidioides* (*1*). Emergomycosis manifests after dissemination to the lungs and skin; it is associated with 50% mortality. Most cases of emergomycosis have been reported in persons with HIV from South Africa infected with *E. africanus*, the DNA of which has been amplified from soil there (*2*). Emergomycosis from *E. orientalis* or *E. canadensis* infection has been identified in limited geographic areas. 

In contrast, *E. pasteurianus* infections have been widely documented in Asia, Europe, and South Africa (Appendix Table 3) (*2*). *E. pasteurianus* infections were first described in 1998 in a patient in Italy with HIV infection and skin lesions (*3*). The isolate was initially placed in the genus *Emmonsia* because of the similarity of the ribosomal large subunit genes. The new genus *Emergomyces* was suggested by Dukik et al. to distinguish fungi that produce small yeasts in host tissues, comparable to *Histoplasma* instead of the adiaspores found in *Emmonsia* (*4*). We report a case of *E.* *pasteurianus* infection in a patient in Uganda with HIV infection.

A 38-year-old woman from Rwanda sought treatment in Uganda for a 3-month history of disseminated skin lesions, nodules, papules, and ulcers (Figure). A chest radiograph revealed no signs of disease. The woman was living in southwestern Uganda, working as a trader. She reported no travel except for a short visit to Dubai 5 years earlier. She had also been diagnosed with HIV 5 years earlier. She was treated for HIV with zidovudine, lamivudine, and nevirapine. CD4 lymphocyte count was 140 cells/µL. HIV viral load testing was not performed. 

**Figure:**
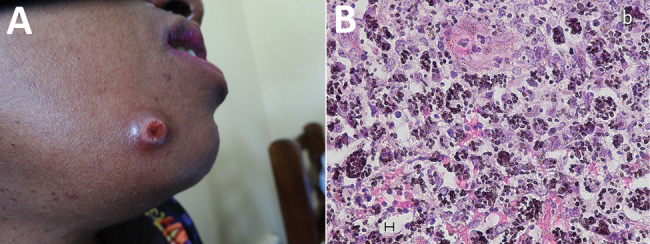
Imaging from investigation of emergomycosis in a 38-year-old woman from Rwanda with HIV infection living in Uganda. A) Skin lesion on face. B) Histopathology of skin biopsy specimen (Grocott stain) showing multiple budding yeast cells (2–3 µm), mostly in clusters. Scale bar indicates 5 µm.

A skin biopsy was taken, but fungal isolation was not performed because laboratorians lacked the necessary equipment. As therapy for emergomycosis, experts suggest amphotericin B, which was not available for the patient, followed by oral triazoles (*2*). The patient was started on fluconazole (400 mg 1×/d) for suspected cutaneous cryptococcosis. Histopathology showed narrow budding yeast cells (2–4 µm) (Figure). Because skin lesions increased during 6 weeks of fluconazole and antiretroviral treatments, treatment was changed to itraconazole (400 mg 2×/d). Lesions decreased markedly within 8 weeks, which we considered the key finding suggesting treatment response. No follow-up data are available beyond this point. As reported by Dukik et al., in vitro resistance testing of *Emergomyces* documents activity of itraconazole, voriconazole, and posaconazole, but not fluconazole (*5*). 

In Germany, DNA was extracted from the formalin-fixed, paraffin-embedded (FFPE) skin biopsy as previously described (*6*). Fungal DNA was amplified by 2 broad-range PCR assays targeting a region of the 28S and the internal transcribed spacer (ITS) 2 regions of the fungal ribosomal RNA genes. Sanger sequencing of the PCR amplicons revealed 365-bp and 273-bp sequences (Appendix). A BLAST search (https://blast.ncbi.nlm.nih.gov/Blast.cgi) revealed *Paracoccidioides lutzii* (98.6% pairwise identity) and *E. pasteurianus* (98.9% pairwise identity) to be the closest matches for the 28S and ITS2 amplicons. Because no generally accepted pairwise identity break points for fungal species identification are available and sequence data for the region amplified by the 28S assay are lacking for many fungi, we sequenced the amplicons of the 2 broad-range PCR assays from fungi of the family *Ajellomycetaceae* and of the species *Coccidioides immitis* (Appendix Table 2). Phylogenetic analysis of the concatenated sequences of both broad-range PCR assays suggested that the patient was infected with *E. pasteurianus* or a closely related species (Appendix Figure). 

Identification of fungi from pathology blocks may be used to investigate the etiology of mycosis and define endemic regions of fungal pathogens. However, species identification by histopathology is limited and the optimal molecular identification strategy remains to be defined. Amplification of fungal DNA from FFPE tissue is restricted by amplicon length, PCR inhibition, an excess of host DNA, and contaminating fungal DNA (*7*). The broad-range assays we used were introduced to amplify fungal DNA from an excess of host DNA. They have been successfully applied on FFPE tissue before (*6*,*8*). 

The ITS2 assay targets a diverse, noncoding region well represented in public databases. However, variable amplicon length (200–300 bp) suggests that detection limits may vary for different fungi and phylogenetic analysis may be impaired. In contrast, the 28S assay amplifies a more conserved coding region (330–350 bp). Whereas identification of a genus may be achieved, species resolution within a genus may not be possible and sequences of this region are underrepresented in public databases (*4*,*6*). 

Our results suggest that emergomycosis is more widespread and genetically diverse than previously documented. This case suggests that using broad-range fungal PCR assays with specific PCR assays to target prevalent pathogens may be a successful approach for identifying fungal etiology from pathology blocks and defining the epidemiology of emergomycosis and related infections.

Appendix Additional information about disseminated emergomycosis in a person with HIV infection, Uganda.
