# Supplementary material for: Disseminated Emergomycosis in a Person with HIV Infection, Uganda
Source: Emerg Infect Dis. 2019 Sep;25(9):1750–1. doi: 10.3201/eid2509.181234 (PMC6711225; doi:10.3201/eid2509.181234)
Supplement: Appendix — Additional information about disseminated emergomycosis in a person with HIV infection, Uganda. [file 18-1234-Techapp-s1.pdf]

Article DOI: https://doi.org/10.3201/eid2509.181234

Disseminated Emergomycosis in a Person with HIV Infection, Uganda

Appendix

Methods

Handling of the tissue sample

Sections of 5 µm were cut from a pathology block of the skin biopsy. Aliquots of three sections were placed in Eppendorf Biopur tubes (Eppendorf AG, https://corporate.eppendorf.com/en/). The first and last sections were stained by Grocott's methenamine silver stain for microscopy to document the presence of fungal elements in the tissue. This research was approved by the institutional review board at Goethe University in Frankfurt, Germany (375/10).

DNA extraction and PCR assays

DNA extraction and PCR setup were performed in a laminar air flow hood to avoid contamination by ubiquitous fungi. DNA was extracted from an aliquot of 3 cuts (5µm each) after paraffin was removed from the sample by octane. DNA extraction from the FFPE skin biopsy was done using the MasterPure Yeast DNA extraction kit (Biozym, https://www.biozym.com/site/313/biozym.aspx) with some modifications, as previously described (*1*). First, the deparaffinized tissue sections were placed with 550µl of yeast cell lysis buffer into tubes containing silicon carbide sharps (BioSpec Products, https://biospec.com/) and shaken for 60 seconds at 5 m/s using a FastPrep 120 machine (MP Biomedicals, https://www.mpbio.com/) to allow for a mechanical disruption of the tissue and fungal elements in the tissue. Second, the initial incubation step of the extraction kit in lysis buffer was carried out at 90° for 3 hours because previous experiments suggested increased amounts of amplifiable DNA after application of thermal energy (*2*). Next, following the manufacturer's instructions for DNA precipitation, the samples were exposed to 750µl ice cold isopropanol 100% and incubation at −20°C for 60 minutes. DNA was dissolved in 75µl Triton X 0.1% and stored at

4°C until the PCR assay (*1*,*2*). All qPCR assays were performed using an Applied Biosystems (https://www.thermofisher.com/us/en/home/brands/applied-biosystems.html) 7500 real-time instrument as described previously (*1*). All assays were run for 45 cycles. Fungal PCRs were run in duplicates.

An internal amplification control excluded PCR inhibition. Successful DNA extraction was demonstrated by detection of human DNA by qPCR (target 18s rRNA Gene). A specific qPCR assay for *Histoplasma*, targeting a variable region of ribosomal RNA genes conserved in *Histoplasma* (target ITS1 region), failed to amplify DNA from the sample (*3*). Therefore, two broad-range fungal qPCR assays targeting the 28S- (primer 28S10f-28S12r), and ITS2 region (primer 5,8Sf-28S1r) of the ribosomal RNA genes were used. Both assays were designed to amplify a broad range of fungal DNA in human samples (i.e., primer was designed to bind regions conserved among pathogenic fungi but show mismatches with human DNA) (*4*). PCR primers are described in Appendix Table 1. Further information on performing these assays have been reported previously (*1*).

Fungal DNA was amplified by both broad-range PCR assays. Amplicons of the broad-range assays were purified using the ExoSap-IT-kit (USB-products, https://www.thermofisher.com/us/en/home/life-science/pcr/united-states-biochemical-usb.html) and sequenced bidirectionally. Mastermix negative controls and extraction negative controls, performed as described previously, ruled out fungal DNA contamination (*1*).

Phylogenetic Analysis

Fungal DNA of fungi related to *Emergomyces* was extracted as reported for the tissue samples after cultivation on potato dextrose agar (Appendix Table 2). Fungal DNA was amplified using both broad-range PCR assays as reported for the tissue sample and sequenced accordingly. The phylogenetic tree was inferred from the combined 28S and ITS-2 sequences amplified from the tissue block and reference strains using the maximum likelihood method based on the Tamura 3-parameter model (Appendix Figure 1) (*5*). The model was determined based on the model with the lowest Bayesian information scores as determined using the finding model program in MEGA7 (*6*). Bootstrap was run with 1000 resampling.

References

1. Rickerts V, Khot PD, Myerson D, Ko DL, Lambrecht E, Fredricks DN. Comparison of quantitative real time PCR with sequencing and ribosomal RNA-FISH for the identification of fungi in formalin fixed, paraffin-embedded tissue specimens. BMC Infect Dis. 2011;11:202. https://doi.org/10.1186/1471-2334-11-202

2. Rickerts V, Khot PD, Ko DL, Fredricks DN. Enhanced fungal DNA-extraction from formalin-fixed, paraffin-embedded tissue specimens by application of thermal energy. Med Mycol. 2012;50:667–72. https://doi.org/10.3109/13693786.2012.665613

3. Simon S, Veron V, Boukhari R, Blanchet D, Aznar C. Detection of *Histoplasma capsulatum* DNA in human samples by real-time polymerase chain reaction. Diagn Microbiol Infect Dis. 2010;66:268–73. https://doi.org/10.1016/j.diagmicrobio.2009.10.010

4. Khot PD, Ko DL, Fredricks DN. Sequencing and analysis of fungal rRNA operons for development of broad-range fungal PCR assays. Appl Environ Microbiol. 2009;75:1559–65. https://doi.org/10.1128/AEM.02383-08

5. Tamura K. Estimation of the number of nucleotide substitutions when there are strong transition-transversion and G+C-content biases. Mol Biol Evol. 1992;9:678–87.

6. Kumar S, Stecher G, Tamura K. Mega 7: Molecular Evolutionary Genetics Analysis version 7 for bigger datasets. Mol Biol Evol. 2016;33:1870–4. https://doi.org/10.1093/molbev/msw054

7. Gori SD, Gueho E, Huerre M, Lofaro A, Parenti M, Dupont B. Cutaneous disseminated mycosis in a patient with AIDS due to a new dimorphic fungus. J Mycol Med. 1998;8:57–63.

8. Pelegrín I, Alastruey-Izquierdo A, Ayats J, Cuenca-Estrella M, Cabellos C. A second look at *Emmonsia* infection can make the difference. Transpl Infect Dis. 2014;16:519–20. https://doi.org/10.1111/tid.12214

9. Lavergne R-A, Kandel-Aznar C, Khatchatourian L, Garcia-Hermoso D, Jeddi F, Boutoille D, et al. *Emmonsia pasteuriana*: Une cause rare d’infection fongique chez l’immunodéprimé. J Mycol Med. 2017;27:e7–8. https://doi.org/10.1016/j.mycmed.2017.04.025

10. Malik R, Capoor MR, Vanidassane I, Gogna A, Singh A, Sen B, et al. Disseminated *Emmonsia pasteuriana* infection in India: a case report and a review. Mycoses. 2016;59:127–32. https://doi.org/10.1111/myc.12437

11. Feng P, Yin S, Zhu G, Li M, Wu B, Xie Y, et al. Disseminated infection caused by *Emmonsia pasteuriana* in a renal transplant recipient. J Dermatol. 2015;42:1179–82. https://doi.org/10.1111/1346-8138.12975

12. Tang XH, Zhou H, Zhang XQ, Han JD, Gao Q. Cutaneous disseminated emmonsiosis due to *Emmonsia pasteuriana* in a patient with cytomegalovirus enteritis. JAMA Dermatol. 2015;151:1263–4. https://doi.org/10.1001/jamadermatol.2015.1792

13. Dukik K, Muñoz JF, Jiang Y, Feng P, Sigler L, Stielow JB, et al. Novel taxa of thermally dimorphic systemic pathogens in the *Ajellomycetaceae* (Onygenales). Mycoses. 2017;60:296–309. https://doi.org/10.1111/myc.12601

Appendix Table 1. Primer used to amplify fungal DNA from FFPE tissue

qPCR	Primer name	Sequence (5′-3′)	Ref
Histoplasma ITS1	Hc-ITS 167f<br>Hc-ITS 229r	AACGATTGGCGTCTGAGCAT<br>GAGATCCGTTGTTGAAAGTTTTGA	3
Broad-range ITS2	5.8Sf<br>28S1r	GTGAATCATCGARTCTTTGAAC<br>TATGCTTAAGTTCAGCGGGTA	4
Broad-range 28S	28S10f<br>28S12r	GACATGGGTTAGTCGATCCTA<br>CCTTATCTACATTRTTCTATCAAC	4

Appendix Table 2. Generated 28S and ITS2 sequences of fungi

Fungus	Identifier	28 S	ITS2
Histoplasma capsulatum	RKI 12–0644	MH682062	MH682095
Blastomyces percursus	CBS 17–139878	MH682055	MH682088
Blastomyces dermatitidis	ATCC-18188	MH682053	MH682086
Paracoccidioides lutzii	ATCC-MYA-826	MH682061	MH682094
Blastomyces parvus	CBS 139881	MH682054	MH682087
Emergomyces africanus	CBS 136260	MH682056	MH682089
Emergomyces pasteurianus	CBS 101426	MH682058	MH682091
Emergomyces orientalis	CBS 124587	MH682057	MH682090
Emergomyces europaeus	CBS 102456*<br>(GHP 1427, UAMH 10427)	MH682059	MH682092
Coccidioides immitis	RKI 06–0091	MH682060	MH682093
This report	RKI 17–0923	MH686148	MH686147

*obtained from G. Haase (14)

Appendix Table 3. Case reports of emergomycosis caused by *Emergomyces pasteurianus*

Author	Country, Year	Preexisting conditions	Clinical presentation	Accession no. (ITS)
Gori (*7*)	Italy, 1998	AIDS	Disseminated (s)	HF563671
Pelegrin (*8*)	Spain, 2014	AIDS, liver transplantation	Disseminated (s,p)	Not reported
Lavergne (*9*)	France or Georgia, 2017	AIDS	Pulmonary	Not reported
Malik (*10*)	India, 2016	AIDS	Disseminated (s, p)	KR150770
Feng (*11*)	China, 2015	Renal transplantation	Disseminated (s, p)	KT155632<br>KP730695<br>KP730694
Tang (*12*)	China, 2015	Glucocorticoid therapy	Disseminated (s)	KP260922
Dukik (*13*)	South Africa, 2017	AIDS	Disseminated (s)	KY195962
This report	Uganda or Rwanda, 2018	AIDS	Disseminated (s)	MH686147

s: skin, p: pulmonary, ITS: internal transcribed spacer

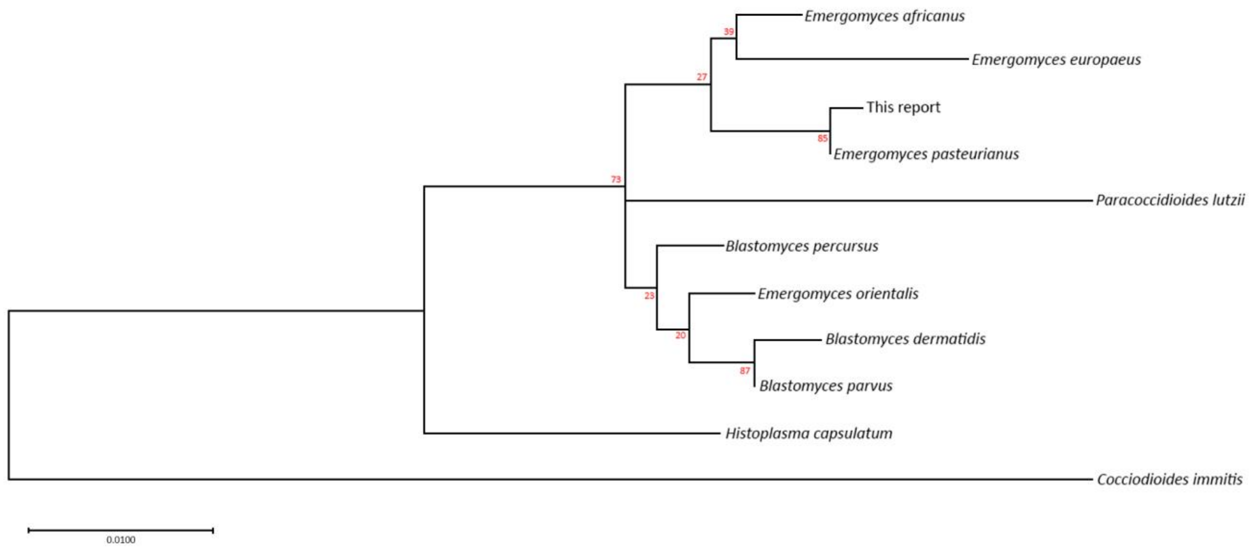

Appendix Figure. Phylogenetic tree of the combined ITS and 28S sequences amplified from the tissue block containing the skin biopsy and sequences from reference strains. A maximum likelihood method using the Tamura 3-parameter substitution model was used based on the lowest Bayesian information scores as determined using the finding model program in MEGA7. Bootstrap was run with 1000 resampling. The percentage of trees in which the associated taxa clustered together is shown next to the branches. The tree is drawn to scale, with branch lengths measured in the number of substitutions per site. There were a total of 551 positions in the final dataset.
